# Plasma and Urinary Phenolic Profiles after Acute and Repetitive Intake of Wild Blueberry

**DOI:** 10.3390/molecules21091120

**Published:** 2016-08-25

**Authors:** Rodrigo P. Feliciano, Geoffrey Istas, Christian Heiss, Ana Rodriguez-Mateos

**Affiliations:** Division of Cardiology, Pulmonology and Vascular Medicine, Medical Faculty, University of Düsseldorf, 40225 Düsseldorf, Germany; Feliciano@med.uni-duesseldorf.de (R.P.F.); geoffrey.istas@med.uni-duesseldorf.de (G.I.); christian.heiss@med.uni-duesseldorf.de (C.H.)

**Keywords:** (poly)phenols, blueberry, acute, chronic, plasma, urine, inter-individual variability

## Abstract

Recent studies have shown that blueberries may have cardiovascular and cognitive health benefits. In this work, we investigated the profile of plasma and urine (poly)phenol metabolites after acute and daily consumption of wild blueberries for 30 days in 18 healthy men. The inter-individual variability in plasma and urinary polyphenol levels was also investigated. Blood samples were collected at baseline and 2 h post-consumption on day 1 and day 30. Twenty-four-hour urine was also collected on both days. A total of 61 phenolic metabolites were quantified in plasma at baseline, of which 43 increased after acute or chronic consumption of blueberries over one month. Benzoic and catechol derivatives represented more than 80% of the changes in phenolic profile after 2 h consumption on day 1, whereas hippuric and benzoic derivatives were the major compounds that increased at 0 and 2 h on day 30, respectively. The total (poly)phenol urinary excretion remained unchanged after 30 days of wild blueberry intake. The inter-individual variability ranged between 40%–48% in plasma and 47%–54% in urine. Taken together, our results illustrate that blueberry (poly)phenols are absorbed and extensively metabolized by phase II enzymes and by the gut microbiota, leading to a whole array of metabolites that may be responsible for the beneficial effects observed after blueberry consumption.

## 1. Introduction

Evidence on the health benefits of polyphenol-rich foods has been accumulating for the last decade [1,2]. Blueberries, in particular, are getting increasing attention due to their potential cardiovascular and cognitive health benefits [3,4,5,6,7,8,9,10]. However, the mechanisms of action (MOA) and the bioactive molecules have not been identified. As a starting point to understand the biological health effects of (poly)phenols, it is necessary to understand which metabolites circulate in plasma at the time of the observed biological effects. In most of the published clinical studies investigating health benefits of blueberries, the (poly)phenol metabolites present in the plasma or urine are not reported. Besides implications for MOA, understanding the metabolome of blueberry (poly)phenols is important in terms of compliance and safety in clinical trials. Furthermore, previous work investigating the absorption, metabolism, and excretion of berry (poly)phenols in humans focused exclusively on the analysis of structurally related anthocyanin metabolites. The fact that the latter were found in plasma at low nanomolar or picomolar levels [11,12,13] led to the conclusion that anthocyanins were poorly absorbed and had very low bioavailability. However, more recent evidence suggest that phenolic acid metabolites derived from the breakdown or metabolism of anthocyanins are the major compounds circulating in blood and urine after anthocyanin consumption, with concentrations up to 100 times higher than the anthocyanin parent compounds [8,11,14,15,16]. Besides being rich sources of anthocyanins, blueberries are also rich in other phenolic compounds such as proanthocyanidins, chlorogenic acids, and flavonols [17,18,19]. Consequently, these compounds will greatly contribute to the pool of phenolic metabolites present in plasma and urine after blueberry consumption.

We have previously reported the bioavailability of blueberry (poly)phenols in plasma after acute intake using enzymatic treatment [7,8,20]. Twenty-three plasma metabolites were found including flavonols, benzoic, hippuric, cinnamic, phenylacetic, and phenylproprionic acids. However, information about the levels of circulating (poly)phenols arising from phase II metabolism is lost when using this approach. To our knowledge, a comprehensive investigation on the metabolism and excretion of blueberry (poly)phenols has not been conducted so far. It is also unknown whether chronic intake of blueberry has an impact on the profile and concentration of phenolic metabolites. Thus, the aim of this work is to identify and quantify plasma and urine (poly)phenol metabolites after acute and chronic intake of blueberry using authentic standards including phase II metabolites in healthy men. The inter-individual variability in plasma and urinary phenolic metabolites was also investigated.

## 2. Results

### 2.1. Characteristics Study Population

The clinical characteristics of the subjects participating in the study are detailed in Table 1. All values are within the normal range for healthy individuals.

### 2.2. (Poly)phenol Content of Blueberry Intervention

Volunteers consumed 11 g of freeze-dried blueberry powder (equivalent to 100 grams of fresh blueberry) dissolved in water twice daily. Each drink contained a total of 302 ± 6 mg of total (poly)phenols. Of these, 150 ± 3 mg were anthocyanins, 64 ± 1 mg chlorogenic acids, 49 ± 2 mg flavanol oligomers (between trimers and decamers), 3 ± 0 mg flavonols dimers, 4 ± 0 mg flavanol monomers, 31 ± 0 mg flavonols, 1 ± 0 mg benzoic, and cinnamic acids.

### 2.3. Plasma Blueberry (Poly)phenol Metabolites after Acute and Repetitive Intake

A total of 61 phenolic metabolites were quantified in plasma at baseline on day 1, of which 43 increased after acute or chronic consumption of blueberries over one month (Table 2). These were benzoic acids, phenylacetic acids, propionic acids, pyrogallols, catechols, hipuric acids, cinnamic acids, flavonols, and valerolactone derivatives.

**Figure 1:**
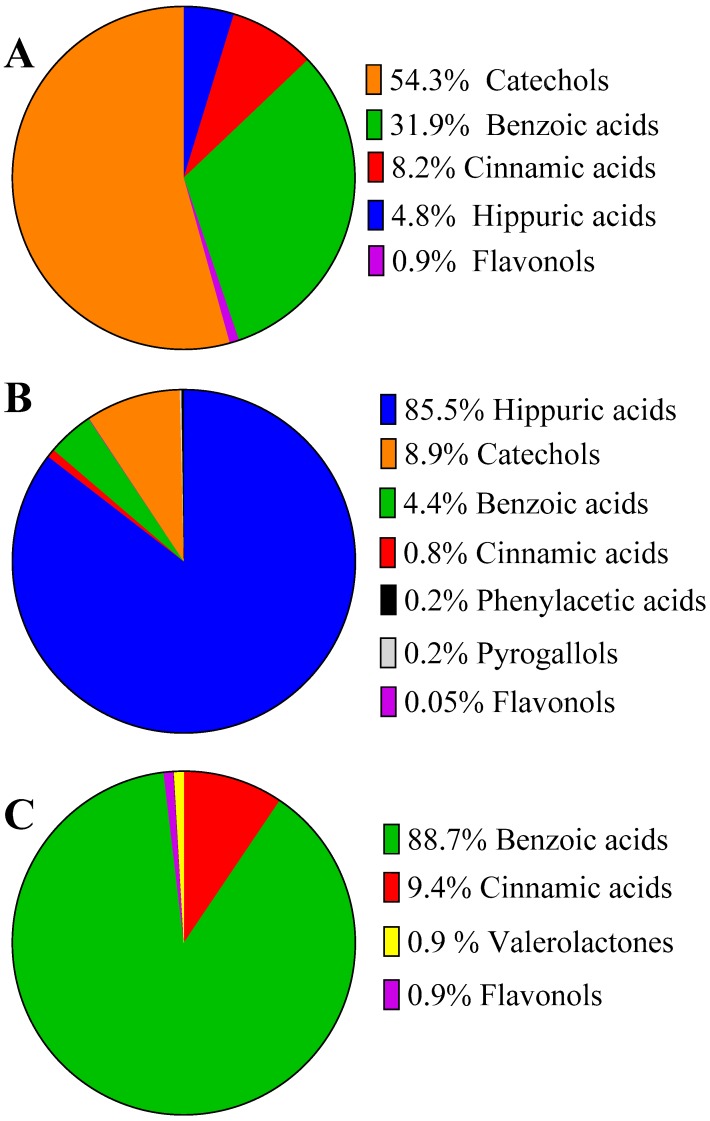
Distribution of (poly)phenols classes which exhibited an increase in plasma concentrations after: (**A**) 2 h of blueberry consumption on day 1 (acute intake); (**B**) one month daily supplementation (chronic); and (**C**) 2 h post-consumption on day 30 (acute on chronic).

At 2 h after blueberry consumption, plasma concentration of 19 compounds increased, including one catechol, four flavonols, two hippuric, six benzoic, and six cinnamic acids, but only chlorogenic acid and quercetin 3-*O*-β-d-glucuronide reached statistical significance with respect to baseline (*p* = 0.003 and 0.004, respectively). The largest changes in plasma were detected for catechol-*O*-sulfate (Δ = 531 ± 346 nM), benzoic acid (Δ = 138 ± 74 nM) and vanillic acid (Δ = 96 ± 70 nM). Overall, catechols, benzoic, and cinnamic acids were responsible for 95% of the increases in plasma phenolic compounds after acute blueberry consumption (Figure 1A).

When comparing the baseline plasma concentrations on day 1 with day 30 (chronic or repetitive intake), 38 compounds increased their levels, including 15 cinnamic acid, eight benzoic acids, four hippuric acids, three phenylacetic acids, two pyrogallols, two catechols, and one benzaldehyde. Of these, eight compounds displayed a significantly higher plasma concentration: dihydrocaffeic acid 3-*O*-sulfate (*p* = 0.004), catechol-O-sulfate (*p* = 0.016), vanillic acid (*p* = 0.005), hippuric acid (*p* = 0.021), 3-hydroxyhippuric acid (*p* = 0.0013), isoferulic acid 3-*O*-β-d-glucuronide (*p* = 0.033), benzoic acid (*p* = 0.046), and 2,5-dihydroxybenzoic acid (0.048). The most abundant compound detected in plasma was by far hippuric acid, which almost double its concentration after the 30-day chronic intake (17.4 µM and 32.5 µM, respectively, Δ = 15 ± 5 µM), followed by catechol-*O*-sulfate (Δ = 1.4 ± 0.5 µM) and 3-hydroxyhippuric acid (Δ = 0.4 ± 0.1 µM), justifying 94% of the positive changes detected (Figure 1B). Minor contributions could be attributed to benzoic acids, followed by cinnamic acids and to a lesser extent to phenylacetic acids, pyrogallols, and flavonols.

On day 30, 19 (poly)phenols increased their plasma concentration at 2 h after consumption (acute + chronic intake, Table 2). The increases were statistically significant for chlorogenic acid (*p* = 0.002), 4-methylgallic acid-3-*O*-sulfate (*p* < 0.001) and quercetin 3-*O*-β-d-glucuronide (*p* = 0.006). The highest plasma level variations were detected for 2,3-dihydroxybenzoic acid (Δ = 589 ± 1882 nM), vanillic acid (Δ = 81 ± 85 nM),s and chlorogenic acid (Δ = 40 ± 20 nM), which accounted for almost all the plasma increases with only minor contributions of valerolactones and flavonols (0.9% each) (Figure 1C). Interestingly, as reflected in Figure 1, the major changes in plasma phenolic metabolites were different for acute, chronic, and acute on chronic intake.

### 2.4. Urinary Excretion of Blueberry (Poly)phenols after Acute and Repetitive Intake

A total of 62 phenolic metabolites were detected in urine after blueberry consumption, with a very similar profile as detected in plasma, except for gallic acid which was only detected in urine (Table 3). Of these, 29 phenolic metabolites increased their 24 h urinary excretion at day 30 when compared to excretion at day 1 of the intervention although without statistical significance (Table 3). (Poly)phenols that presented an increase after repetitive intake were mainly catechols, benzoic, hippuric, cinnamic, and phenylacetic acids. Urinary recovery was in average 16% ± 2% and 17% ± 2% for days 1 and 30, respectively.

### 2.5. Inter-Individual Variability in Plasma and Urinary Levels of Blueberry (Poly)phenol Metabolites

**Figure 2:**
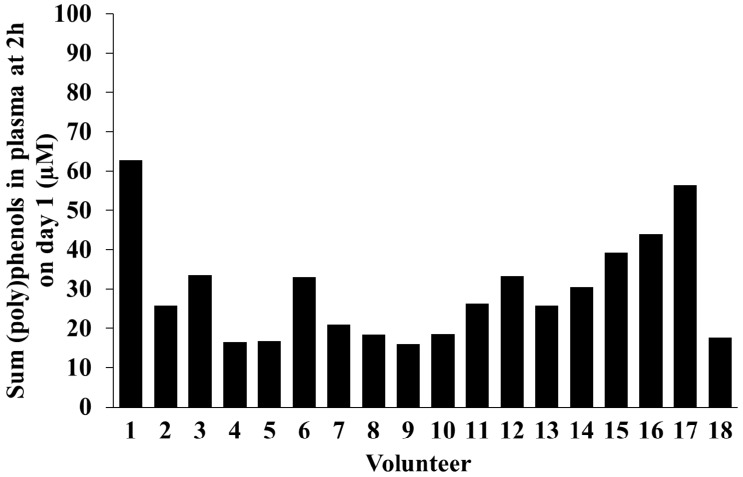
Total (poly)phenols concentration (µM) at 2 h post consumption of wild blueberries at day 1 (*n* = 18).

The sum of total (poly)phenol metabolites quantified in plasma at 2 h post-consumption on day 1 is presented in Figure 2 for each volunteer. The average total plasma concentration was 30 µM, ranging between 16 µM and 63 µM, with a coefficient of variation (CV) of 44%.

**Figure 3:**
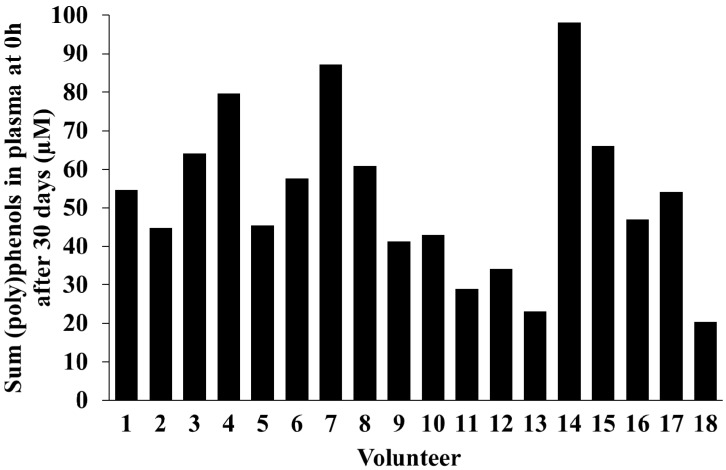
Total (poly)phenols concentration (µM) at 0 h after daily consumption of wild blueberries for 30 days (*n* = 18).

One month after blueberry daily consumption the average plasma concentration of the sum of (poly)phenols at baseline was 53 µM. The minimum and maximum concentrations detected were 20 and 98 µM, respectively (Figure 3), with a CV = 40%. At 2 h post-consumption (acute + chronic) the sum of (poly)phenols quantified in plasma ranged from 10 to 109 µM and the CV was 48%.

The 24 h total (poly)phenol urinary excretion values were calculated for both days 1 and 30 (Table 3). The CV% was 47% and 54% for days 1 and 30, respectively (Table 3). The recovery of (poly)phenols in urine also registered considerable large inter-individual variability. At day 1 the urinary recovery rates varied between 4% and 25%, while these values ranged from 2% to 41% at day 30.

Interindividual variability was also assessed in terms of classes of (poly)phenols. Catechols and benzoic acids showed the lowest inter-individual variability in plasma (36%–58% and 50%–63%, respectively), whereas in urine benzaldehydes, phenylacetic and benzoic acids were the compounds excreted with less variability (47%–69%, 50%–54% and 40%–71%, respectively) (Table 4). Propionic acids, pyrogallols, and valerolactones were the (poly)phenols with the highest inter-individual variability, either in plasma or urine. As far as the sum of (poly)phenols detected in either plasma or excreted in urine is concerned, the inter-individual variability was lower in plasma (40%–48%) than in urine (47%–54%).

## 3. Discussion

In this work, we report for the first time the profile of phase II and gut microbial metabolites of blueberry (poly)phenols in plasma and urine of healthy volunteers after acute and repetitive intake. A total of 61 and 62 metabolites were found in plasma and urine, respectively, of which 19 were found to increase at 2 h post consumption and 38 after one month daily supplementation of wild blueberry. We report here a statistically significant increase at 2 h post consumption of quercetin 3-*O*-β-d-glucuronide and chlorogenic acid, which exhibited a two- and three-fold increase, respectively. Although 17 other compounds increased their concentration at 2 h compared to baseline it is likely that the T_max_ of these compounds was not reached at 2 h. (Poly)phenols as *p*-coumaric acid, caffeic acid 3-*O*-β-d-glucuronide, ferulic acid 4-*O*-β-d-glucuronide, isoferulic acid 3-*O*-β-d-glucuronide reach their T_max_ as early as 1 h post consumption while catechol-*O*-sulfate, benzoic, isovanillic, 2-hydroxyhippuric, and 3-hydroxyhippuric acids reach their T_max_ at least 6 h after cranberry intake [21]. The most abundant compound at 2 h post consumption was hippuric acid, followed by 2,3-dihydroxybenzoic acid, phenylacetic acid, catechol-*O*-sulfate, and isoferulic acid. Hippuric and isoferulic acids increases in plasma concentration have been associated with blueberry intake [7,8,20] and catechol-*O*-sulfate has been reported after consumption of mixed berries [22] or cranberry juice [21].

One month after daily consumption of blueberry, 38 compounds increased their fasted plasma level when compared to the first day of the intervention. To the best of our knowledge this is the first report describing increases in plasma concentration after sustained intake of wild blueberry. Significant increases were detected for eight compounds, including catechols, namely catechol-*O*-sulfate, three benzoic acids, two hippuric acids and two phase II cinnamic acid metabolites. Metabolites such as benzoic, 2,5-dihydroxybenzoic, and vanillic acids can be present in blueberry but they are also products of the gut microbiota or flavonoid metabolism [23]. Phase II metabolites as catechol-*O*-sulfate, dihydrocaffeic acid 3-*O*-sulfate, isoferulic acid 3-*O*-β-d-glucuronide were also significantly increased after one month which suggest that blueberry (poly)phenols may selectively alter the metabolizing capacity of sulfotransferases and glucuronosyltransferases; however, not all the phase II metabolites showed a significant increase. The efficiency of the gut microbiota to metabolize polyphenols may also have been affected, as all metabolites increasing concentration after one month could be derived from gut microbial metabolism. Hippuric acid was the biggest contributor of the (poly)phenol pool of circulating metabolites, accounting to approximately 15 µM (86%). These results are in agreement with previous works which showed that hippuric acid was significantly associated with bilberry consumption after a 12-week intervention [24] and with a high-(poly)phenol intake for eight weeks [25]. Our findings are extremely pertinent as the higher levels of hippuric acid and other (poly)phenols could justify, in part, the improvements detected after daily intake of blueberry in cardiovascular health and memory [3,4,5,6,10,26]. However, we cannot discard the possibility that these metabolites are coming from the intake of the blueberry drink the evening before or even from other (poly)phenol sources in the diet. A limitation of the current work is that plasma was collected only at one time point (2 h). Moreover, the level of some metabolites, even when individuals are properly fasted and followed a low polyphenol diet for 24 h, can be high and mask increases deriving from a (poly)phenol intervention. Limited data is available regarding the bioaccumulation of polyphenols after chronic intake. In agreement with our data, daily intake of varied berries for eight weeks show significant increases in several plasma (poly)phenol metabolites, including quercetin, *p*-coumaric acid, caffeic acid, protocatechuic acid, vanillic and homovanillic acid, 3-hydroxyphenylpropionic acid, and 3-(3-hydroxyphenyl)propionic acid [27]. Previous works in which volunteers were given three different doses of quercetin over two weeks resulted in increased plasma levels of quercetin metabolites [28]. A 12 week supplementation with cocoa flavanols also increased fasted plasma levels of epicatechin and valerolactone metabolites [29]. In contrast, supplementation of an elderberry extract for 12-weeks showed no significant increases in plasma levels of anthocyanin metabolites [30]. Whether or not (poly)phenol disposition is fast and bioaccumulation negligible after chronic exposure needs further investigation. The evidence available suggest that the effects are likely not relevant in terms of toxicity, as no adverse effects were reported in those studies.

In this work, we also report for the first time the urinary profile after acute and chronic intake of wild blueberries. Hippuric acids were the major (poly)phenols excreted in urine, followed by cinnamic, benzoic, phenylacetic acids, and valerolactones. Although almost half of the compounds registered an increase in 24 h-urine when comparing day 30 with day 1, these increases were discrete and not statistically significant, leading to a total polyphenol excretion of 95 and 101 mg which corresponds to 16% and 17% of total urinary recovery, respectively. Our results are in agreement with Ferrars et al. which also did not observe any significant differences in excretion of anthocyanins from elderberry when comparing acute and chronic interventions [30]. However, Koli et al., found significant increases in urinary levels of quercetin, *p*-coumaric and 3-hydroxyphenylacetic acid after eight week berry consumption, with no differences in other phenolic metabolites [27]. Although further work is needed, the available data suggest that chronic consumption of berries do not greatly affect the urinary excretion of polyphenols. Studies with other (poly)phenol rich foods have reported significant increases in some urinary catechin metabolites after six and 12 weeks of green tea supplementation [31], suggesting that the bioaccumulation of (poly)phenols depends on their chemical composition.

The bioavailability of plant food bioactives may be dependent on intrinsic (e.g., age, sex, ethnicity, disease, genetic polymorphisms, etc.) and extrinsic factors (e.g., dose, food matrix, dietary habits, etc.) [32,33,34,35,36,37,38]. In this study, a large inter-individual variability was seen after wild blueberry intake not only at 2 h in plasma but also in 24 h (poly)phenol urinary excretion. When considering classes of (poly)phenols we reported a minimum and maximum CV in plasma of 36% and 228% for catechols and phenylacetic acids, respectively, at 2 h post-consumption on day 1. These magnitudes of variation are in agreement with a previous report that showed a CV in terms of C_max_ of 22% for 2,5-dihydroxybenzoic acid and 225% for phenylacetic acid [20], after intake of a similar amount (766 mg) of wild blueberry (poly)phenols. Other authors have reported high plasma inter-individual variability in response to berry intake. Pimpao et al. quantified (poly)phenol sulfates with C_max_ CV between 133% for pyrogallol-*O*-1-sulfate (652 ± 328 nM) and 238% for vanillic acid-*O*-sulfate (1345 ± 1310 nM) after the consumption of mixed berries [22]. After intake of ^13^C-labelled anthocyanins, the C_max_ CV of (poly)phenol sulfates varied between 76% for protocatechuic acid-4-sulfate (1244 ± 333 nM) and 152% for isovanillic acid-3-sulfate (822 ± 557 nM) [14]. Large inter-individual variability was also reported after consumption of raspberries with CV of C_max_ oscillating between 33% for ferulic acid-4-*O*-β-d-glucuronide (18 ± 2 nM) and 161% for urolithin B-3-*O*-glucuronide (23 ± 14 nM) [39]. The high inter-individual variability of urolithins has led to stratification of individuals according to their metabotype [40] and can be an interesting approach to decrease the large variability observed in human intervention studies with (poly)phenols and other bioactive food components. Stratification of different genotypes in terms of protein efflux transporters of phase II metabolites has showed significant differences in isoflavones plasma levels [41].

Interestingly, when considering the sum of all (poly)phenols concentration after 2 h post consumption of wild blueberry, the CV ranged between 44% and 48% which is in agreement with a CV of 49% of the AUC of the sum of 23 metabolites reported after blueberry intake [20]. Our results suggest that the pool of total circulating (poly)phenol metabolites has a lower inter-individual variability then plasma concentration of individual compounds. The inter-individual variability was also evident when analyzing the 24 h urine samples. The urinary excretion was approximately 17% either acutely or chronically but in some individuals urinary recovery rates as low as 2% and as high as 34% were observed. Previous works have showed that urinary excretion of (poly)phenols can vary between 0.05% and 6.3% after strawberry consumption, or between 0.21% and 7.6% following raspberry intake [42].

One of the factors that may explain part of the variability observed in this work is differences in the gut microbiota, which composition is affected by (poly)phenol intake [43,44], but also varies with diet, ethnicity, among other factors. Future works should focus on stratification of individuals based on gut microbiota composition as recently done in the context of nutrition [45] and health diagnostics [46]. Age and sex stratification may also be important factors explaining inter-individual variability, although a recent study suggests that the absorption, metabolism and excretion of cocoa flavanols is not age dependent [37].

In conclusion, our current data illustrate that blueberry (poly)phenols are absorbed and extensively metabolized by phase II enzymes and by the gut microbiota, leading to a whole array of metabolites that may be responsible for the beneficial effects observed after blueberry consumption. Our results also indicate that chronic consumption of blueberry may lead to modest changes in the metabolizing capacity of enzymes and gut microbiota, as an increase in plasma phenolic metabolites was seen after daily consumption of blueberry. However, no differences were seen in the total urinary excretion of polyphenols suggesting that sustained consumption of blueberry does not induce sufficient bioaccumulation to enhance systemic toxicity. The characterization of blueberry related metabolites in plasma may help to understand the biological health effects of (poly)phenols.

## 4. Materials and Methods

### 4.1. Intervention Study Subjects

Eighteen healthy male volunteers from 18 to 70 years were recruited from the University of Düsseldorf and surrounding area. Health was ascertained by a routine clinical physical exam and specific cardiovascular history performed by a cardiovascular specialist. Manifest cardiovascular disease, including coronary artery disease, cerebrovascular disease, and peripheral artery disease, diabetes mellitus, acute inflammation, terminal renal failure, malignancies, and heart rhythm other than sinus were exclusion criteria.

### 4.2. Human Study Design

This study was part of a double blind, parallel randomized controlled trial conducted to investigate the vascular effects of freeze-dried wild blueberry consumption in a population of healthy men comparing daily wild blueberry ingestion over one month with a control/placebo drink. Informed consent was obtained and subjects were randomized to the treatments. We investigated the plasma and urinary phenolic profile of 18 healthy men after daily consumption of 11 g of freeze-dried blueberry powder (equivalent to 100 g of fresh blueberries) dissolved in water twice per day. Plasma samples were taken at baseline and at 2 h post-acute consumption on day 1 and after one month of daily consumption of blueberry at baseline and at 2 h, as well. Urine was collected between 0–12 h and 12–24 h on day 1 and one month after blueberry supplementation.

Volunteers were instructed not to alter their usual dietary or fluid intake. Those selected for the study were asked to refrain from the following for 24 h prior to the study: consumption of polyphenol-rich foods including fruits, vegetables, cocoa, chocolate, coffee, tea, and wine, participating in vigorous exercise (>3 × 20 min/week) and consuming more than 168 g of alcohol (any form) per week. Compliance to the diet and lifestyle restrictions was determined via a 24 h-dietary recall and via interview.

This study was conducted according to the guidelines laid down in the Declaration of Helsinki and all procedures involving human subjects were approved by the University of Düsseldorf Research Ethics Committee (ref: 4306). The study was also registered with the National Institutes of Health (NIH)-randomized trial records held on the NIH ClinicalTrials.gov website (NCT02520830).

### 4.3. Blueberry Test Drinks

The Wild Blueberry Association of North America (WBANA) supplied the freeze-dried wild blueberry. Each sachet of blueberry (11 g of freeze-dried blueberry powder, dissolved in 500 mL of water) contained 363 mg of total polyphenols, so the daily polyphenol consumption was 726 mg.

### 4.4. Plasma and Urine Collection

The blood samples collected in EDTA/heparin tubes were spun (1700× *g*; 15 min; 4 °C) immediately after collection. Samples for (poly)phenol analysis were spiked with 2% formic acid. Ascorbic acid was added to urine containers (3.75 g/2 L container) prior to urine collection. Urine containers were kept in an opaque cool bag with ice blocks at all times during the 24 h collection. The total volume of urine excreted by each volunteer was measured and an aliquot was taken and acidified with formic acid until pH 2.5 was achieved. All samples were aliquoted and frozen at −80 °C until analysis.

### 4.5. UPLC-Q-TOF MS Analysis of Plasma (Poly)phenols

Plasma and urinary analysis of polyphenol metabolites was performed using microelution solid phase extraction coupled with ultra high-performance liquid chromatography quadrupole time of flight coupled to mass spectrometry (UPLC-Q-TOF MS) and authentic standards for quantification as previously described [21]. Urinary recovery (%) was calculated by normalizing the concentration measured in urine (nM) in terms of volume excreted (mL) during each collection time point. The nmoles excreted were further converted into µg in order to be able to calculate the urinary recovery. This parameter was determined by dividing the sum of total (poly)phenols excreted in urine (mg) in 24 h by the total (poly)phenols consumed per day (726 mg). Due to failure in collecting one time point (12–24 h) on day 30, volunteers 9 and 17 were not taken into account for the urinary excretion calculations (*n* = 16).

### 4.6. Statistical Analysis

Average values of results are presented as mean values and their standard error of means. Comparison between plasma concentrations at 2 h post consumption with baseline on day 1 (acute) and baseline after 30 days of blueberry supplementation with baseline at day 0 (chronic) were done by one-way ANOVA or Wilcoxon test, depending if the distributions had equal or unequal variances, respectively. Analyses were computed with Graph Pad Prism 6 and JMP Pro 11.

## Figures and Tables

**Table 1:** Characteristics study population (*n* = 18).

Mean ± SD	
Age (years)	33 ± 18
BMI (kg/m^2^)	24 ± 3
Systolic blood pressure (mm·Hg)	125 ± 10
Diastolic blood pressure (mm·Hg)	73 ± 9
Heart rate (bpm)	65 ± 9
Total cholesterol (mg/dL)	175 ± 33
Triglycerides (mg/dL)	90 ± 49
HDL-cholesterol (mg/dL)	55 ± 10
LDL-cholesterol (mg/dL)	114 ± 33
Fasting plasma glucose (mg/dL)	84 ± 14

**Table 2:** Plasma (poly)phenol concentration at 0 and 2 h post-consumption of wild blueberries (*n* = 18) after acute (day 1) and repetitive intake (day 30). Compounds which had statistically higher plasma concentrations at 2 h when compared to 0 h on day 1 (acute) are marked with ^a^. Statistical significant plasma concentrations when comparing 0 h at day 30 with 0 h on day 1 (chronic) were marked with ^b^ and statistical significance at 2 h on day 30 when compared to 0 h on the same day (acute + chronic) are marked with ^c^.

	Plasma Concentration (nM)			
	Day 1		Day 30	
	0 h	2 h	0 h	2 h
	**Benzoic acid Derivatives**			
Benzoic acid	794 ± 56	932 ± 63	981 ± 72 ^b^	926 ± 47
2-Hydroxybenzoic acid	80 ± 22	131 ± 60	103 ± 15	106 ± 15
3-Hydroxybenzoic acid	17 ± 3	13 ± 2	21 ± 5	17 ± 3
4-Hydroxybenzoic acid	27 ± 8	23 ± 6	18 ± 4	18 ± 3
2,3-Dihydroxybenzoic acid	8891± 1550	7430 ± 1323	8070 ± 1210	8658 ± 1642
2,4-Dihydroxybenzoic acid	13 ± 5	11 ± 3	25 ± 7	18 ± 3
2,5-Dihydroxybenzoic acid	71 ± 10	54 ± 6	101 ± 10 ^b^	94 ± 11
Protocatechuic acid	22 ± 6	18 ± 6	11 ± 4	8 ± 3
Syringic acid	4 ± 1	8 ± 2	9 ± 3	11 ± 3
Vanillic acid	290 ± 48	397 ± 53	644 ± 122 ^b^	730 ± 109
Vanillic acid-4-*O*-sulfate	30 ± 6	29 ± 6	34 ± 6	34 ± 6
Isovanillic acid	429 ± 51	447 ± 58	426 ± 46	430 ± 57
4-Methylgallic acid-3-*O*-sulfate	43 ± 28	48 ± 11	28 ± 7	58 ± 15 ^c^
	**Phenylacetic acid derivatives**			
Homovanillic acid	74 ± 7	52 ± 3	86 ± 14	71 ± 11
Homovanillic acid sulfate	4 ± 1	4 ± 1	6 ± 1	4 ± 1
Phenylacetic acid	2854 ± 1319	2599 ± 1578	1634 ± 603	1130 ± 315
3,4-Dihydroxyphenylacetic acid	91 ± 13	76 ± 9	89 ± 14	87 ± 11
3-Hydroxyphenylacetic acid	142 ± 32	103 ± 25	174 ± 29	152 ± 27
4-Hydroxyphenylacetic acid	333 ± 50	215 ± 31	289 ± 61	230 ± 49
	**Propionic acid derivatives**			
2-(4-hydroxyphenoxy)propionic acid	4 ± 1	3 ± 1	2 ± 0	2 ± 1
	**Benzaldehyde derivatives**			
4-Hydroxybenzaldehyde	62 ± 17	58 ± 16	55 ± 10	46 ± 10
3,4-Dihydroxybenzaldehyde	1 ± 0	1 ± 0	1 ± 0	1 ± 0
	**Pyrogallol derivatives**			
Pyrogallol-*O*-1-sulfate	22 ± 6	15 ± 2	23 ± 5	15 ± 2
Pyrogallol-*O*-2-sulfate	134 ± 60	99 ± 48	104 ± 44	62 ± 23
1-Methylpyrogallol-*O*-sulfate	87 ± 24	57 ± 16	112 ± 36	90 ± 31
2-Methylpyrogallol-*O*-sulfate	61 ± 18	58 ± 18	29 ± 7	22 ± 4
	**Catechol derivatives**			
Catechol-*O*-sulfate	1228 ± 231	1759 ± 179	2726 ± 440 ^b^	1565 ± 195
4-Methylcatechol-*O*-sulfate	693 ± 104	468 ± 81	860 ± 250	775 ± 234
	**Hippuric acid derivatives**			
Hippuric acid	17,445 ± 3746	12,367 ± 2464	32545 ± 5252 ^b^	29,644 ± 5587
2-Hydroxyhippuric acid	5 ± 2	9 ± 4	8 ± 2	8 ± 2
3-Hydroxyhippuric acid	290 ± 72	320 ± 84	708 ± 159 ^b^	416 ± 87
4-Hydroxyhippuric acid	77 ± 12	59 ± 8	76 ± 12	64 ± 8
α-Hydroxyhippuric acid	485 ± 66	385 ± 43	539 ± 82	463 ± 62
	**Cinnamic acid derivatives**			
Cinnamic acid	22 ± 5	22 ± 5	20 ± 4	20 ± 6
Caffeic acid	7 ± 2	7 ± 2	6 ± 2	7 ± 2
Caffeic acid 3-*O*-β-d-glucuronide	2 ± 1	2 ± 1	4 ± 3	3 ± 1
Caffeic acid 4-*O*-β-d-glucuronide	1 ± 0	1 ± 0	2 ± 2	2 ± 1
Dihydrocaffeic acid	24 ± 6	17 ± 4	27 ± 6	23 ± 5
Dihydrocaffeic acid 3-*O*-sulfate	53 ± 12	41 ± 11	104 ± 28 ^b^	73 ± 15
Dihydrocaffeic acid 3-*O*-β-d-glucuronide	9 ± 1	8 ± 1	11 ± 2	10 ± 2
Ferulic acid	6 ± 2	6 ± 2	7 ± 2	6 ± 1
Ferulic acid 4-*O*-glucuronide	156 ± 40	182 ± 32	195 ± 76	223 ± 67
Ferulic acid 4-*O*-sulfate	90 ± 33	106 ± 40	74 ± 26	80 ± 17
Dihydroferulic acid	100 ± 23	72 ± 24	111 ± 22	74 ± 15
Dihydroferulic acid 4-*O*-sulfate	154 ± 26	96 ± 19	138 ± 29	90 ± 17
Dihydroferulic acid 4-*O*-β-d-glucuronide	116 ± 23	76 ± 16	124 ± 31	100 ± 25
Isoferulic acid	1941 ± 348	1633 ± 312	1842 ± 377	1686 ± 319
Isoferulic acid 3-*O*-sulfate	19 ± 3	18 ± 2	22 ± 5	20 ± 4
Isoferulic acid 3-*O*-β-d-glucuronide	38 ± 10	38 ± 8	74 ± 13 ^b^	77 ± 16
Dihydro isoferulic acid 3-*O*-sulfate	102 ± 64	73 ± 39	44 ± 11	40 ± 8
Dihydroisoferulic acid 3-*O*-β-d-glucuronide	8 ± 2	6 ± 2	9 ± 2	8 ± 2
*m*-Coumaric acid	1 ± 0	1 ± 0	1 ± 0	1 ± 0
*o*-Coumaric acid	1 ± 0	1 ± 0	1 ± 0	1 ± 0
*p*-Coumaric acid	3 ± 0	4 ± 1	4 ± 1	4 ± 1
Sinapic acid	11 ± 2	7 ± 1	8 ± 1	7 ± 1
Chlorogenic acid	22 ± 10	60 ± 18 ^a^	20 ± 6	63 ± 22 ^c^
	**Flavonol derivatives**			
Kaempferol	64 ± 17	68 ± 17	71 ± 22	75 ± 23
Kaempferol-3-*O*-β-d-glucuronide	1 ± 0	1 ± 0	2 ± 2	2 ± 0
Quercetin	32 ± 8	35 ± 9	28 ± 7	28 ± 7
Quercetin-3-*O*-β-d-glucuronide	3 ± 1	5 ± 1 ^a^	6 ± 1	9 ± 2 ^c^
	**Valerolactone derivatives**			
(4*R*)-5-(3′-hydroxyphenyl)-γ-valerolactone-4′-*O*-sulfate	62 ± 26	31 ± 10	60 ± 16	67 ± 25

**Table 3:** Urinary excretion (24 h) after acute (day 1) and repetitive intake (day 30) of wild blueberries. Data is expressed as average ± SEM (µg). The % recovery (bold values at bottom) represent the percentage of the total amount of (poly)phenols ingested in the blueberry drink that were excreted in urine. No statistical significances were found between day 1 and day 30.

Total 24 h Urinary Excretion (µg)	Day 1	Day 30
**Benzoic Acid Derivatives**		
Benzoic acid	204 ± 49	286 ± 115
2-Hydroxybenzoic acid	50 ± 30	18 ± 10
3-Hydroxybenzoic acid	22 ± 7	22 ± 5
4-Hydroxybenzoic acid	519 ± 97	397 ± 60
2,3-Dihydroxybenzoic acid	6590 ± 1351	6079 ± 727
2,4-Dihydroxybenzoic acid	54 ± 13	61 ± 15
2,5-Dihydroxybenzoic acid	668 ± 119	757 ± 106
Protocatechuic acid	284 ± 52	217 ± 47
Syringic acid	86 ± 21	76 ± 20
Vanillic acid	264 ± 88	219 ± 36
Vanillic acid-4-*O*-sulfate	392 ± 103	407 ± 147
Isovanillic acid	68 ± 10	69 ± 13
Gallic acid	2 ± 1	1 ± 0
4-Methylgallic acid-3-*O*-sulfate	194 ± 56	149 ± 50
**Phenylacetic acid derivatives**		
Homovanillic acid	939 ± 175	941 ± 143
Homovanillic acid sulfate	37 ± 9	48 ± 19
Phenylacetic acid	318 ± 50	429 ± 129
3,4-Dihydroxyphenylacetic acid	219 ± 39	168 ± 29
3-Hydroxyphenylacetic acid	900 ± 130	1230 ± 188
4-Hydroxyphenylacetic acid	1910 ± 284	1592 ± 199
**Propionic acid derivatives**		
2-(4-hydroxyphenoxy)propionic acid	38 ± 19	39 ± 15
**Benzaldehyde derivatives**		
4-Hydroxybenzaldehyde	2 ± 0	1 ± 0
3,4-Dihydroxybenzaldehyde	25 ± 4	20 ± 2
**Pyrogallol derivatives**		
Pyrogallol-*O*-1-sulfate	22 ± 6	19 ± 6
Pyrogallol-*O*-2-sulfate	154 ± 41	92 ± 34
1-Methylpyrogallol-*O*-sulfate	71 ± 19	74 ± 31
2-Methylpyrogallol-*O*-sulfate	48 ± 15	35 ± 12
**Catechol derivatives**		
Catechol-*O*-sulfate	325 ± 69	456 ± 138
4-Methylcatechol-*O*-sulfate	842 ± 127	874 ± 181
**Hippuric acid derivatives**		
Hippuric acid	55,827 ± 7000	62,840 ± 8552
2-Hydroxyhippuric acid	651 ± 308	555 ± 297
3-Hydroxyhippuric acid	5729 ± 938	6242 ± 1119
4-Hydroxyhippuric acid	480 ± 116	397 ± 114
α-Hydroxyhippuric acid	4887 ± 1198	3857 ± 1052
**Cinnamic acid derivatives**		
Cinnamic acid	2 ± 1	2 ± 1
Caffeic acid	18 ± 2	20 ± 3
Caffeic acid 3-*O*-β-d-glucuronide	142 ± 25	148 ± 37
Caffeic acid 4-*O*-β-d-glucuronide	67 ± 12	60 ± 12
Dihydrocaffeic acid	46 ± 9	74 ± 14
Dihydrocaffeic acid 3-*O*-sulfate	824 ± 194	1032 ± 244
Dihydrocaffeic acid 3-*O*-β-d-glucuronide	105 ± 20	114 ± 20
Ferulic acid	10 ± 3	13 ± 2
Ferulic acid 4-*O*-glucuronide	1942 ± 266	1924 ± 300
Ferulic acid 4-*O*-sulfate	3861 ± 638	3828 ± 597
Dihydroferulic acid	147 ± 44	152 ± 36
Dihydroferulic acid 4-*O*-sulfate	954 ± 158	932 ± 136
Dihydroferulic acid 4-*O*-β-d-glucuronide	427 ± 130	404 ± 79
Isoferulic acid	832 ± 243	889 ± 371
Isoferulic acid 3-*O*-sulfate	310 ± 79	302 ± 74
Isoferulic acid 3-*O*-β-d-glucuronide	290 ± 58	284 ± 55
Dihydro isoferulic acid 3-*O*-sulfate	121 ± 27	184 ± 64
Dihydro isoferulic acid 3-*O*-β-d-glucuronide	152 ± 39	166 ± 47
*m*-Coumaric acid	9 ± 2	15 ± 3
*o*-Coumaric acid	2 ± 0	1 ± 0
*p*-Coumaric acid	3 ±1	2 ± 0
Sinapic acid	60 ± 16	46 ± 14
Chlorogenic acid	370 ± 85	338 ± 102
**Flavonol derivatives**		
Kaempferol	51 ± 13	70 ± 18
Kaempferol-3-*O*-β-D-glucuronide	6 ± 2	4 ± 1
Quercetin	51 ± 11	140 ± 76
Quercetin-3-*O*-β-D-glucuronide	19 ± 4	24 ± 8
**Valerolactone derivatives**		
(4*R*)-5-(3′-hydroxyphenyl)-γ-valerolactone-4′-*O*-sulfate	1354 ± 277	1246 ± 292
**Total amount excreted (mg)**	**95 ± 11**	**101 ± 13**
**Recovery (%)**	**16 ± 2**	**17 ± 2**

**Table 4:** Coefficient of variation (%) of (poly)phenols detected in plasma and urine after acute and chronic wild blueberry consumption.

	Plasma				Urine	
	Day 1		Day 30		Day 1	Day 30
	0 h	2 h	0 h	2 h	24 h	24 h
Benzoic acids	63	61	50	62	71	40
Phenylacetic acids	164	228	120	85	50	54
Propionic acids	96	111	105	127	198	158
Benzaldehydes	115	117	78	90	69	47
Pyrogallols	87	133	141	125	77	134
Catechols	52	36	58	45	61	84
Hippuric acids	90	82	66	78	50	57
Cinnamic acids	65	63	79	74	57	65
Flavonols	81	83	91	88	79	151
Valerolactones	182	148	113	157	82	94
**Total (poly)phenols**	**44**	**44**	**40**	**48**	**47**	**54**

## References

[1] Del Rio D., Rodriguez-Mateos A., Spencer J.P., Tognolini M., Borges G., Crozier A. (2013). Dietary (poly)phenolics in human health: Structures, bioavailability, and evidence of protective effects against chronic diseases. Antioxid. Redox Signal..

[2] Rodriguez-Mateos A., Vauzour D., Krueger C.G., Shanmuganayagam D., Reed J., Calani L., Mena P., Del Rio D., Crozier A. (2014). Bioavailability, bioactivity and impact on health of dietary flavonoids and related compounds: An update. Arch. Toxicol..

[3] Johnson S.A., Figueroa A., Navaei N., Wong A., Kalfon R., Ormsbee L.T., Feresin R.G., Elam M.L., Hooshmand S., Payton M.E. (2015). Daily Blueberry Consumption Improves Blood Pressure and Arterial Stiffness in Postmenopausal Women with Pre- and Stage 1-Hypertension: A Randomized, Double-Blind, Placebo-Controlled Clinical Trial. J. Acad. Nutr. Diet..

[4] McAnulty L.S., Collier S.R., Landram M.J., Whittaker D.S., Isaacs S.E., Klemka J.M., Cheek S.L., Arms J.C., McAnulty S.R. (2014). Six weeks daily ingestion of whole blueberry powder increases natural killer cell counts and reduces arterial stiffness in sedentary males and females. Nutr. Res..

[5] Stull A.J., Cash K.C., Champagne C.M., Gupta A.K., Boston R., Beyl R.A., Johnson W.D., Cefalu W.T. (2015). Blueberries improve endothelial function, but not blood pressure, in adults with metabolic syndrome: A randomized, double-blind, placebo-controlled clinical trial. Nutrients.

[6] Basu A., Du M., Leyva M.J., Sanchez K., Betts N.M., Wu M., Aston C.E., Lyons T.J. (2010). Blueberries decrease cardiovascular risk factors in obese men and women with metabolic syndrome. J. Nutr..

[7] Rodriguez-Mateos A., Pino-Garcia R.D., George T.W., Vidal-Diez A., Heiss C., Spencer J.P.E. (2014). Impact of processing on the bioavailability and vascular effects of blueberry (poly)phenols. Mol. Nutr. Food Res..

[8] Rodriguez-Mateos A., Rendeiro C., Bergillos-Meca T., Tabatabaee S., George T.W., Heiss C., Spencer J.P. (2013). Intake and time dependence of blueberry flavonoid-induced improvements in vascular function: A randomized, controlled, double-blind, crossover intervention study with mechanistic insights into biological activity. Am. J. Clin. Nutr..

[9] Whyte A.R., Schafer G., Williams C.M. (2015). Cognitive effects following acute wild blueberry supplementation in 7- to 10-year-old children. Eur. J. Nutr..

[10] Krikorian R., Shidler M.D., Nash T.A., Kalt W., Vinqvist-Tymchuk M.R., Shukitt-Hale B., Joseph J.A. (2010). Blueberry supplementation improves memory in older adults. J. Agric. Food Chem..

[11] De Ferrars R.M., Czank C., Zhang Q., Botting N.P., Kroon P.A., Cassidy A., Kay C.D. (2014). The pharmacokinetics of anthocyanins and their metabolites in humans. Br. J. Pharmacol..

[12] Mazza G., Kay C.D., Cottrell T., Holub B.J. (2002). Absorption of anthocyanins from blueberries and serum antioxidant status in human subjects. J. Agric. Food Chem..

[13] McKay D.L., Chen C.Y.O., Zampariello C.A., Blumberg J.B. (2015). Flavonoids and phenolic acids from cranberry juice are bioavailable and bioactive in healthy older adults. Food Chem..

[14] Czank C., Cassidy A., Zhang Q., Morrison D.J., Preston T., Kroon P.A., Botting N.P., Kay C.D. (2013). Human metabolism and elimination of the anthocyanin, cyanidin-3-glucoside: A (13)C-tracer study. Am. J. Clin. Nutr..

[15] Nurmi T., Mursu J., Heinonen M., Nurmi A., Hiltunen R., Voutilainen S. (2009). Metabolism of berry anthocyanins to phenolic acids in humans. J. Agric. Food Chem..

[16] Vitaglione P., Donnarumma G., Napolitano A., Galvano F., Gallo A., Scalfi L., Fogliano V. (2007). Protocatechuic Acid Is the Major Human Metabolite of Cyanidin-Glucosides. J. Nutr..

[17] Rodriguez-Mateos A., Cifuentes-Gomez T., Tabatabaee S., Lecras C., Spencer J.P. (2012). Procyanidin, anthocyanin, and chlorogenic acid contents of highbush and lowbush blueberries. J. Agric. Food Chem..

[18] Scalzo J., Currie A., Stephens J., Alspach P., McGhie T. (2008). The anthocyanin composition of different Vaccinium, Ribes and Rubus genotypes. Biofactors.

[19] Feliciano R.P., Krueger C.G., Reed J.D. (2015). Methods to determine effects of cranberry proanthocyanidins on extraintestinal infections: Relevance for urinary tract health. Mol. Nutr. Food Res..

[20] Rodriguez-Mateos A., Feliciano R.P., Cifuentes-Gomez T., Spencer J.P.E. (2016). Bioavailability of wild blueberry (poly)phenols at different levels of intake. J. Berry Res..

[21] Feliciano R.P., Boeres A., Massacessi L., Istas G., Ventura M.R., Nunes dos Santos C., Heiss C., Rodriguez-Mateos A. (2016). Identification and quantification of novel cranberry-derived plasma and urinary (poly)phenols. Arch. Biochem. Biophys..

[22] Pimpao R.C., Ventura M.R., Ferreira R.B., Williamson G., Santos C.N. (2015). Phenolic sulfates as new and highly abundant metabolites in human plasma after ingestion of a mixed berry fruit puree. Br. J. Nutr..

[23] Mosele J.I., Gosalbes M.J., Macia A., Rubio L., Vazquez-Castellanos J.F., Jimenez Hernandez N., Moya A., Latorre A., Motilva M.J. (2015). Effect of daily intake of pomegranate juice on fecal microbiota and feces metabolites from healthy volunteers. Mol. Nutr. Food Res..

[24] Hanhineva K., Lankinen M.A., Pedret A., Schwab U., Kolehmainen M., Paananen J., de Mello V., Sola R., Lehtonen M., Poutanen K. (2015). Nontargeted metabolite profiling discriminates diet-specific biomarkers for consumption of whole grains, fatty fish, and bilberries in a randomized controlled trial. J. Nutr..

[25] Vetrani C., Rivellese A.A., Annuzzi G., Adiels M., Boren J., Mattila I., Oresic M., Aura A.M. (2016). Metabolic transformations of dietary polyphenols: Comparison between in vitro colonic and hepatic models and in vivo urinary metabolites. J. Nutr. Biochem..

[26] Schrager M.A., Hilton J., Gould R., Kelly V.E. (2015). Effects of blueberry supplementation on measures of functional mobility in older adults. Appl. Physiol. Nutr. Metab..

[27] Koli R., Erlund I., Jula A., Marniemi J., Mattila P., Alfthan G. (2010). Bioavailability of various polyphenols from a diet containing moderate amounts of berries. J. Agric. Food Chem..

[28] Egert S., Wolffram S., Bosy-Westphal A., Boesch-Saadatmandi C., Wagner A.E., Frank J., Rimbach G., Mueller M.J. (2008). Daily quercetin supplementation dose-dependently increases plasma quercetin concentrations in healthy humans. J. Nutr..

[29] Ottaviani J.I., Balz M., Kimball J., Ensunsa J.L., Fong R., Momma T.Y., Kwik-Uribe C., Schroeter H., Keen C.L. (2015). Safety and efficacy of cocoa flavanol intake in healthy adults: A randomized, controlled, double-masked trial. Am. J. Clin. Nutr..

[30] De Ferrars R.M., Cassidy A., Curtis P., Kay C.D. (2014). Phenolic metabolites of anthocyanins following a dietary intervention study in post-menopausal women. Mol. Nutr. Food Res..

[31] Clarke K.A., Dew T.P., Watson R.E., Farrar M.D., Bennett S., Nicolaou A., Rhodes L.E., Williamson G. (2014). High performance liquid chromatography tandem mass spectrometry dual extraction method for identification of green tea catechin metabolites excreted in human urine. J. Chromatogr. B.

[32] Peng Y.S., Peng Y.M., McGee D.L., Alberts D.S. (1994). Carotenoids, tocopherols, and retinoids in human buccal mucosal cells: Intra- and interindividual variability and storage stability. Am. J. Clin. Nutr..

[33] Borel P., Desmarchelier C., Nowicki M., Bott R. (2015). A Combination of Single-Nucleotide Polymorphisms Is Associated with Interindividual Variability in Dietary beta-Carotene Bioavailability in Healthy Men. J. Nutr..

[34] Borel P., Desmarchelier C., Nowicki M., Bott R., Morange S., Lesavre N. (2014). Interindividual variability of lutein bioavailability in healthy men: Characterization, genetic variants involved, and relation with fasting plasma lutein concentration. Am. J. Clin. Nutr..

[35] Lee M.J., Maliakal P., Chen L., Meng X., Bondoc F.Y., Prabhu S., Lambert G., Mohr S., Yang C.S. (2002). Pharmacokinetics of tea catechins after ingestion of green tea and (−)-epigallocatechin-3-gallate by humans: Formation of different metabolites and individual variability. Cancer Epidemiol. Biomark. Prev..

[36] Actis-Goretta L., Leveques A., Giuffrida F., Romanov-Michailidis F., Viton F., Barron D., Duenas-Paton M., Gonzalez-Manzano S., Santos-Buelga C., Williamson G. (2012). Elucidation of (−)-epicatechin metabolites after ingestion of chocolate by healthy humans. Free Radic. Biol. Med..

[37] Rodriguez-Mateos A., Cifuentes-Gomez T., Gonzalez-Salvador I., Ottaviani J.I., Schroeter H., Kelm M., Heiss C., Spencer J.P. (2015). Influence of age on the absorption, metabolism, and excretion of cocoa flavanols in healthy subjects. Mol. Nutr. Food Res..

[38] Gross G., Jacobs D.M., Peters S., Possemiers S., van Duynhoven J., Vaughan E.E., van de Wiele T. (2010). In vitro bioconversion of polyphenols from black tea and red wine/grape juice by human intestinal microbiota displays strong interindividual variability. J. Agric. Food Chem..

[39] Ludwig I.A., Mena P., Calani L., Borges G., Pereira-Caro G., Bresciani L., Del Rio D., Lean M.E., Crozier A. (2015). New insights into the bioavailability of red raspberry anthocyanins and ellagitannins. Free Radic. Biol. Med..

[40] Tomas-Barberan F.A., Garcia-Villalba R., Gonzalez-Sarrias A., Selma M.V., Espin J.C. (2014). Ellagic acid metabolism by human gut microbiota: Consistent observation of three urolithin phenotypes in intervention trials, independent of food source, age, and health status. J. Agric. Food Chem..

[41] Kato K., Kusuhara H., Kumagai Y., Ieiri I., Mori H., Ito S., Nakai Y., Maeda K., Sugiyama Y. (2012). Association of multidrug resistance-associated protein 2 single nucleotide polymorphism rs12762549 with the basal plasma levels of phase II metabolites of isoflavonoids in healthy Japanese individuals. Pharmacogenet. Genom..

[42] Cerda B., Tomas-Barberan F.A., Espin J.C. (2005). Metabolism of antioxidant and chemopreventive ellagitannins from strawberries, raspberries, walnuts, and oak-aged wine in humans: Identification of biomarkers and individual variability. J. Agric. Food Chem..

[43] Queipo-Ortuno M.I., Boto-Ordonez M., Murri M., Gomez-Zumaquero J.M., Clemente-Postigo M., Estruch R., Cardona Diaz F., Andres-Lacueva C., Tinahones F.J. (2012). Influence of red wine polyphenols and ethanol on the gut microbiota ecology and biochemical biomarkers. Am. J. Clin. Nutr..

[44] Boto-Ordonez M., Urpi-Sarda M., Queipo-Ortuno M.I., Tulipani S., Tinahones F.J., Andres-Lacueva C. (2014). High levels of Bifidobacteria are associated with increased levels of anthocyanin microbial metabolites: A randomized clinical trial. Food Funct..

[45] Selma M.V., Romo-Vaquero M., Garcia-Villalba R., Gonzalez-Sarrias A., Tomas-Barberan F.A., Espin J.C. (2016). The human gut microbial ecology associated with overweight and obesity determines ellagic acid metabolism. Food Funct..

[46] Jalanka J., Salonen A., Fuentes S., de Vos W.M. (2015). Microbial signatures in post-infectious irritable bowel syndrome—Toward patient stratification for improved diagnostics and treatment. Gut Microbes.

